# Omicron BA.2 lineage predominance in severe acute respiratory syndrome coronavirus 2 positive cases during the third wave in North India

**DOI:** 10.3389/fmed.2022.955930

**Published:** 2022-11-02

**Authors:** Kamran Zaman, Anita M. Shete, Shailendra Kumar Mishra, Abhinendra Kumar, Mahendra M. Reddy, Rima R. Sahay, Shailendra Yadav, Triparna Majumdar, Ashok K. Pandey, Gaurav Raj Dwivedi, Hirawati Deval, Rajeev Singh, Sthita Pragnya Behera, Niraj Kumar, Savita Patil, Ashish Kumar, Manisha Dudhmal, Yash Joshi, Aishwarya Shukla, Pranita Gawande, Asif Kavathekar, Nalin Kumar, Vijay Kumar, Kamlesh Kumar, Ravi Shankar Singh, Manoj Kumar, Shashikant Tiwari, Ajay Verma, Pragya D. Yadav, Rajni Kant

**Affiliations:** ^1^Indian Council of Medical Research—Regional Medical Research Centre Gorakhpur (ICMR-RMRC Gorakhpur), Gorakhpur, India; ^2^Maximum Containment Facility, Indian Council of Medical Research—National Institute of Virology Pune (ICMR-NIV Pune), Pune, India

**Keywords:** SARS-CoV-2, COVID-19, omicron, variant of concern (VOC), third wave, whole-genome sequencing (WGS)

## Abstract

**Background:**

Recent studies on severe acute respiratory syndrome coronavirus 2 (SARS-CoV-2) reveal that Omicron variant BA.1 and sub-lineages have revived the concern over resistance to antiviral drugs and vaccine-induced immunity. The present study aims to analyze the clinical profile and genome characterization of the SARS-CoV-2 variant in eastern Uttar Pradesh (UP), North India.

**Methods:**

Whole-genome sequencing (WGS) was conducted for 146 SARS-CoV-2 samples obtained from individuals who tested coronavirus disease 2019 (COVID-19) positive between the period of 1 January 2022 and 24 February 2022, from three districts of eastern UP. The details regarding clinical and hospitalized status were captured through telephonic interviews after obtaining verbal informed consent. A maximum-likelihood phylogenetic tree was created for evolutionary analysis using MEGA7.

**Results:**

The mean age of study participants was 33.9 ± 13.1 years, with 73.5% accounting for male patients. Of the 98 cases contacted by telephone, 30 (30.6%) had a travel history (domestic/international), 16 (16.3%) reported having been infected with COVID-19 in past, 79 (80.6%) had symptoms, and seven had at least one comorbidity. Most of the sequences belonged to the Omicron variant, with BA.1 (6.2%), BA.1.1 (2.7%), BA.1.1.1 (0.7%), BA.1.1.7 (5.5%), BA.1.17.2 (0.7%), BA.1.18 (0.7%), BA.2 (30.8%), BA.2.10 (50.7%), BA.2.12 (0.7%), and B.1.617.2 (1.3%) lineages. BA.1 and BA.1.1 strains possess signature spike mutations S:A67V, S:T95I, S:R346K, S:S371L, S:G446S, S:G496S, S:T547K, S:N856K, and S:L981F, and BA.2 contains S:V213G, S:T376A, and S:D405N. Notably, ins214EPE (S1- N-Terminal domain) mutation was found in a significant number of Omicron BA.1 and sub-lineages. The overall Omicron BA.2 lineage was observed in 79.5% of women and 83.2% of men.

**Conclusion:**

The current study showed a predominance of the Omicron BA.2 variant outcompeting the BA.1 over a period in eastern UP. Most of the cases had a breakthrough infection following the recommended two doses of vaccine with four in five cases being symptomatic. There is a need to further explore the immune evasion properties of the Omicron variant.

## Introduction

Emerging variants of severe acute respiratory syndrome coronavirus 2 (SARS-CoV-2) have been identified since the first coronavirus disease 2019 (COVID-19) infection appeared at the end of December 2019 in Wuhan city of China (1). As of 2 September 2022, SARS-CoV-2 has resulted in over 601,189,435 cases worldwide with 6.47 million deaths, while India has recorded a total of 44,442,507 cases with around 5,27,932 deaths due to COVID-19 of which 23,610 such fatalities were reported from Uttar Pradesh (UP) state alone (2, 3). SARS-CoV-2 is the largest enveloped virus with a single-stranded positive-sense ribonucleic acid (RNA) genome of ∼30 kb. To prioritize global SARS-CoV-2 surveillance and research, the World Health Organization (WHO) has classified SARS-CoV-2 variants into three main categories, namely, variants of concern (VOCs), variants of interest (VOIs), and variants under monitoring. At present, there are five VOCs, including Alpha (B.1.1.7), Beta (B.1.351), Gamma (P.1), Delta (B.1.617.2), and Omicron (B.1.1.529). The fourth variant of concern (VOC), B.1.617 (Delta variant) was identified in December 2020 in Maharashtra, India (4, 5). Until November 2021, the Delta variant was designated as a variant of concern (VOC) by the WHO because of its characteristics in terms of the potential to increase transmissibility, virulence, and ability of the variant to evade the immune response of current vaccines. On 24 November 2021, health authorities in South Africa reported the emergence of a new SARS-CoV-2 variant, B.1.1.529 (Omicron), which was classified as the fifth VOC by the WHO on 26 November 2021 (6).

India had experienced three distinct waves of the COVID-19 epidemic. The first wave of COVID-19 infection began in early March 2020, and the peak of the highest number of cases (93,236) was recorded on September 16, 2020, and had returned to relative normality as of February 2021. Sequencing of the SARS-CoV-2 genome revealed that L, S, G, GR, and GH clades were prevalent during the first wave (7). Unfortunately, by the end of March 2021, the number of patients with COVID-19 began to increase exponentially leading to a second wave that was more devastating than the first. India had the highest number of confirmed cases (391,154) on 8 May 2021. Delta variant (B.1.617) was responsible for this deadly second wave of COVID-19 and was associated with breakthrough infections in the country (8). Previous studies have reported various circulating double-mutant (B.1.617) and triple-mutant strains (B.1.618) of SARS-CoV-2 across different regions of India, which are more pathogenic than the initial strains such as B.1.1.7 (Alpha), B.1.351 (Beta), and P.1 (Gamma) (9). After surviving the first and second waves of the SARS-CoV-2 virus, India was hit by a vicious third wave in January 2022. Of late, a sharp rise in COVID-19 cases and deaths are witnessed across India. Currently, five lineages (BA.1, BA.2, BA.3, BA.4, and BA.5) of the SARS-CoV-2 Omicron VOC are circulating globally (10). The VOC Omicron (B.1.1.529) was responsible for a surge in SARS-CoV-2 cases during the third COVID-19 wave in the country (11). According to the bulletin issued by INSACOG on 11 July 2022, Omicron BA.2 and BA.2.38 have mostly been found in India and BA.2.75 sub-variant has acquired more mutations in spike protein and other genes of the SARS-CoV-2.

The Omicron variant has been associated with serious public health concerns due to its higher binding affinity to the angiotensin-converting enzyme 2 (ACE2) receptor (12), high transmissibility, increased viral load, and efficient immune evasion system. Compared with Delta and its subvariants, the Omicron variant carries more than 30 residue substitutions in the receptor-binding domain (RBD) and N-terminal domain of spike protein and, therefore, raises the concern of more efficient cell entry, immune escape potential, and greater infectivity (13, 14). A multitude of mutations in the Omicron variant RBD results in Omicron being immunologically resistant to antibody-mediated protection compared with the Delta variant. In contrast to the SARS-CoV-2 Delta variant, suboptimal S1/S2 cleavage, and inability to use the cellular protease TMPRSS2, syncytium formation by the SARS-CoV-2 Omicron spike is found substantially impaired, a finding suggesting the lower pathogenicity of Omicron (15). The first death with the Omicron variant was reported in Rajasthan in December 2021. New sub-lineages of the SARS-CoV-2 virus Omicron variant have been identified, BA.1 sub-lineage has taken over the COVID-19 landscape, and currently, BA.2 sub-lineage has become the dominant variant circulating in India (16).

Uttar Pradesh is the most populous state in India, and its emergence as an industrial hub and proximity to the border with Nepal makes it vulnerable to migration. UP also witnessed the third wave of SARS-CoV-2 infections in the month of January 2022, with a steep increase in cases followed by a downfall at the end of February 2022. As cases of COVID-19 continue to rise across the country in late December 2021, UP immediately imposed a night curfew from 11 p.m. to 5 a.m. in the state. Besides the curfew, other COVID-related protocols and restrictions were also implemented, including bans on gatherings of more than 200 people, cultural and religious events, etc.^1^ A previous study from eastern UP has provided comprehensive information on SARS-CoV-2 variants and distinct lineage/clades in the first and second waves (17). Existing research on the clinical and genomic characteristics of Omicron cases is limited in India (18–20). This study was conducted to gain a deeper understanding of SARS-CoV-2 mutant strains circulation and their impact on clinical outcomes in the Deoria, Gorakhpur, and Maharajganj districts of UP during the third wave of the pandemic. This study provides a true genomic picture of different SARS-CoV-2 variants and hotspot mutations in all non-structural and structural genes that will aid in examining vaccine efficacy against evolving SARS-CoV-2 and public health planning to control COVID-19 infection.

## Materials and methods

### Sample acquisition

ICMR-Regional Medical Research Centre Gorakhpur (ICMR-RMRC GKP) is a major testing center for SARS-CoV-2 samples from Gorakhpur and its surrounding districts in the eastern UP region of India. A total of 63,977 naso/oropharyngeal (N/OP) samples were collected by the Integrated Disease Surveillance Project (IDSP) team from the districts of Deoria and Maharajganj districts of UP and a tertiary care medical facility (AIIMS, Gorakhpur) from 1 January 2022, to 24 February 2022, and were subjected to testing at our center. Of the 63,977 samples tested, 1,892 samples tested positive for SARS-CoV-2 by real-time PCR (rRT-PCR).

A subset of these samples was subjected to whole-genome sequencing (WGS) to understand the circulating variants. The primary inclusion criteria for the selection of samples for WGS were SARS-CoV-2-positive samples with a cycle threshold value (Ct value) < 30 in rRT-PCR. Hereby, a total of 147 positive samples fulfilling the above criteria were selected and subsequently packed in triple layer packing on dry ice according to the International Air Transport Association (IATA) protocol and transported to the ICMR-National Institute of Virology, Pune, for WGS to identify lineages of the sequences.

### Sociodemographic and clinical course details

The basic demographic details [age, gender, healthcare worker (yes/no), and district] were extracted in Microsoft Excel from the ICMR COVID-19 data portal.^2^ The details regarding vaccination, travel, symptoms, comorbidity, history of previous COVID-19 infection, hospitalized status, and recovery from COVID-19 were captured through telephonic interviews after obtaining verbal informed consent. COVID-19 breakthrough infection was defined as the detection of any COVID-19 infection occurring ≥ 14 days after receiving all recommended doses of either of the vaccine(s) available in the program.

### Statistical analysis

The data were entered in Microsoft Excel and analyzed using IBM SPSS Statistics for Windows, version 24 (IBM Corp., Armonk, NY, USA). Age was expressed as a mean with standard deviation (SD). All categorical variables including variants were expressed as the frequency with percentage. The time between the vaccination and infection in the third wave was reported as median days with an interquartile range (IQR). The case distribution map was generated by using Epi Info 7.2 software program (CDC, Atlanta, USA).

### Ribonucleic acid extraction and whole-genome sequencing

Viral RNA was extracted from N/OP specimens using a MagMAX™ viral pathogen nucleic acid isolation kit (Thermo Fisher Scientific, Waltham, MA, USA). An automated method of KingFisher Flex (Thermo Fisher Scientific, Waltham, MA, USA) magnetic particle processor for high-throughput nucleic acid extraction was used, following the instructions of the manufacturer. Nucleic acid was eluted with 50 μl of elution buffer. N/OP swabs were screened for E and RDRP genes using real-time RT PCR. The extracted RNA of samples with a Ct value < 30 was used further to generate genomic libraries for sequencing using the CovidSeq RUO test kit (Illumina Inc., USA). In brief, the steps involved in the preparation of libraries are fragmentation, adapter ligation, and amplification. The CovidSeq libraries were quantified using the KAPA library quantification kit (Kapa Biosystems, Roche Diagnostics Corporation, USA). The quantified libraries were normalized and loaded onto the Illumina MiniSeq platform for sequencing.

The paired-end FASTQ files generated from the MiniSeq machine were analyzed on the CLC Genomics Workbench, version 21.0.4 (CLC, Qiagen, Hilden, Germany). A reference-based assembly using SARS-CoV-2 isolate Wuhan-Hu-1 (accession no.: NC_045512.2) was used to retrieve the SARS-CoV-2 sequences deposited in the public repository of Global Initiative on Sharing Avian Influenza Data (GISAID) database.

### Phylogenetic analysis

Evolutionary analysis was performed with the sequences obtained from this study along with the previously reported sequences from UP retrieved from the GISAID database. Based on the quality of sequencing results, a total of 146 sequences out of the 147 samples were used to create a cladogram. The sequences were aligned using the CLC Genomics Workbench and manually checked for correctness. The nucleotide variations and amino acid substitutions were annotated. A neighbor-joining (NJ) phylogenetic tree using the Tamura three-parameter model was built using a bootstrap replication of 1,000 cycles that was performed to assess the statistical robustness of the generated phylogenetic tree.

## Results

### Sociodemographic and clinical characteristics

The clinical and sociodemographic characteristics of the 147 individuals who were infected with SARS-CoV-2 infection during the third wave in eastern UP are described in Table 1 and Supplementary Table 1. The mean (SD) age was 33.9 (13.1) years, male patients formed 108 (73.5%) of the cases, healthcare workers were 34 (23.1%), and the district Maharajganj contributed to 61 (41.4%) cases.

**TABLE 1:** Sociodemographic, clinical, and genomic characteristics of sequenced severe acute respiratory syndrome coronavirus 2 (SARS-CoV-2)-positive cases during the third wave (01 January–24 February 2022) in eastern Uttar Pradesh (UP) (*N* = 147).

Characteristics	Number (%)
Age in years (Mean ± SD)	33.9 ± 13.1
**Gender**	
Female	39 (26.5)
Male	108 (73.5)
**District**	
Deoria	43 (29.3)
Gorakhpur	43 (29.3)
Maharajganj	61 (41.0)
**Healthcare worker**	
Yes	34 (23.1)
No	113 (76.9)
**Variants of Concern (VOC) among genome retrieved (*n* = 146)**	
BA.1	9 (6.2)
BA.1.1	4 (2.7)
BA.1.1.1	1 (0.7)
BA.1.1.7	8 (5.5)
BA.1.17.2	1 (0.7)
BA.1.18	1 (0.7)
BA.2	45 (30.8)
BA.2.10	74 (50.7)
BA.2.12	1 (0.7)
B.1.617.2	2 (1.3)
**Type of vaccine (*n* = 98)**	
BBIBP-CorV	3 (3.1)
ChAdOx1 nCoV-19	78 (79.5)
BBV152	4 (4.1)
Not vaccinated	13 (13.3)
**Vaccination status (*n* = 98)**	
1^st^ + 2^nd^ + Precautionary dose	9 (9.2)
1^st^ and 2^nd^ dose	70 (71.4)
Only 1^st^ dose	6 (6.1)
Not vaccinated	13 (13.3)
**Travel history (*n* = 98)**	
Present	30 (30.6)
Absent	68 (69.4)
**Symptom status (*n* = 98)**	
Present	79 (80.6)
Absent	19 (19.4)
Fever	70 (71.4)
Cough	26 (26.5)
Running nose	50 (51.0)
Sore throat	56 (57.1)
Body ache	34 (34.7)
Headache	16 (16.3)
Breathing shortness	9 (9.2)
Loss of taste	7 (7.1)
**Comorbid condition (*n* = 98)**	
Yes*	7 (7.1)
No	91 (92.9)
**History of COVID-19 in past (*n* = 98)**	
Yes	16 (16.3)
No	82 (83.7)

*Includes four cases of diabetes, one case each of hypertension, tuberculosis, and bronchial asthma.

Of 147 cases, we were able to contact 98 cases (66.7) telephonically to get details regarding clinical course and vaccination status. Reasons for not contacting included wrong contact details or the mobile phone being out of coverage area or switched off. There was no refusal to participate among those contacted through telephone. Of the 98 cases who were contacted telephonically, 30 (30.6%) gave a history of travel (domestic/international), 16 (16.3%) reported having been infected with COVID-19 previously, and 7 were having at least one comorbidity. Of the 98 cases, 79 (80.6%) reported having any symptoms with most of them having fever (71.4%) followed by a sore throat (57.1%) and running nose (51.0%). None of the cases were hospitalized, and all were reported to recover from infection.

Most of them had taken vaccination for COVID-19 with 13 (13.3%) reporting not taking any vaccine. Among those who were vaccinated with two doses of the COVID-19 vaccine (*n* = 79), 73 (92.4%) cases had a breakthrough infection (including 26 healthcare workers), with the median (IQR) duration of time to infection from the second dose being 187 (94–293) days. Eight cases who were infected in the third wave had received a precautionary dose with a median (IQR) duration of time to infection from the precautionary dose to be 10 (6.5–23) days.

### WGS data and phylogenetic analysis

Out of 147 samples, reference-based mapping led to the retrieval of 146 genomic sequences with a genome coverage of ≥98%. The percentage of relevant mapped reads had a median (IQR) value of 94.92 (88.9–97.7). Details of the genome retrieval data of the SARS-CoV-2 sequence in this study are provided with GISAID accession IDs in Supplementary Table 2. The SARS-CoV-2 representative sequences VOC/VOI downloaded from the GISAID database^3^ were also used for analysis along with sequences retrieved in this study. The sequences were aligned using the CLC Genomics Workbench. The aligned file was manually checked in MEGA 7.0 software. A neighbor-joining (NJ) phylogenetic tree was constructed by MEGA 7.0 software from the coding region of the SARS-CoV-2 genome using the Tamura 3-parameter model along with gamma distribution as the rate variation parameter. A bootstrap replication of 1,000 cycles was performed to assess the statistical robustness of the generated phylogenetic tree.

**FIGURE 1:**
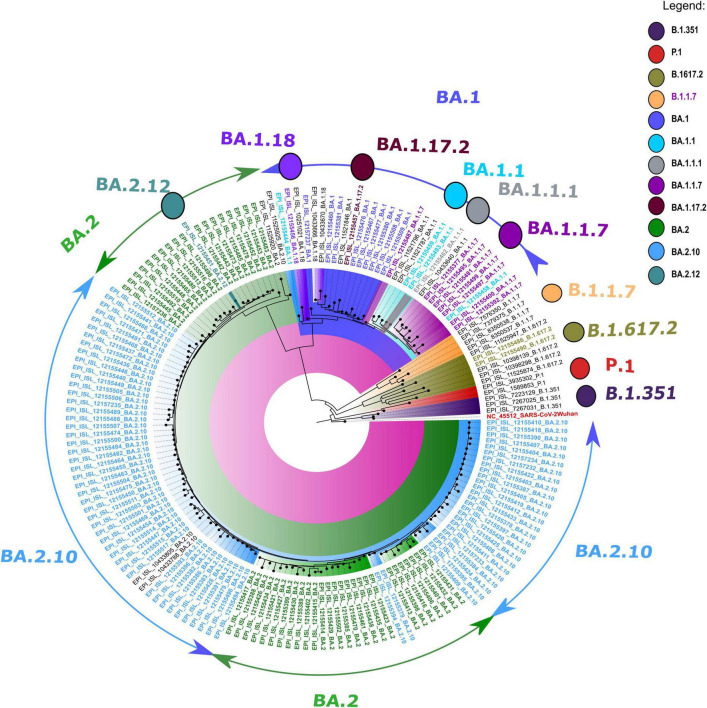
A neighbor-joining (NJ) tree from severe acute respiratory syndrome coronavirus 2 (SARS-CoV-2) sequences was generated using a Tamura 3-parameter model with gamma distribution and a bootstrap replication of 1,000 cycles. The whole-genome sequences of other SARS-CoV-2 variants deposited in GISAID used in this study are marked in black, and the reference isolate of Wuhan-HU-1 (accession no: NC 045512) is marked in red color.

The retrieved sequences were checked in Pangolin software^4^ to identify the specific lineages. The majority of the sequences belonged to the Omicron variant, with BA.1 (*n* = 09), BA.1.1 (*n* = 04), BA.1.1.1 (*n* = 01), BA.1.1.7 (*n* = 08), BA.1.17.2 (*n* = 01), BA.1.18 (*n* = 01), BA.2 (*n* = 45), BA.2.10 (*n* = 74), BA.2.12 (*n* = 01), and B.1.617.2 (*n* = 2) lineages. The NJ tree showed a distinct cluster of Omicron lineages with a clear bifurcation of VOC/VOI (Figure 1). A shift in the circulating lineage from BA.1 to BA.2 was noted over the period time from 01 January 2022 to 24 February 2022. The overall Omicron BA.2 lineage was observed in 79.5% of women (31/39) and 83.2% of men (89/107).

### Variant analysis of severe acute respiratory syndrome coronavirus 2 sequences

All the sequences used in the phylogenetic analysis showed Omicron pangolin lineage except two sequences with Delta lineage B.1.617.2. The CLC genomics was used to identify the mutational changes in the sub-lineages of the Omicron variant. During mutational patterns analysis, we have observed that with the lapse of time, some signature mutations escape out of the spike gene of SARS-CoV-2.

**FIGURE 2:**
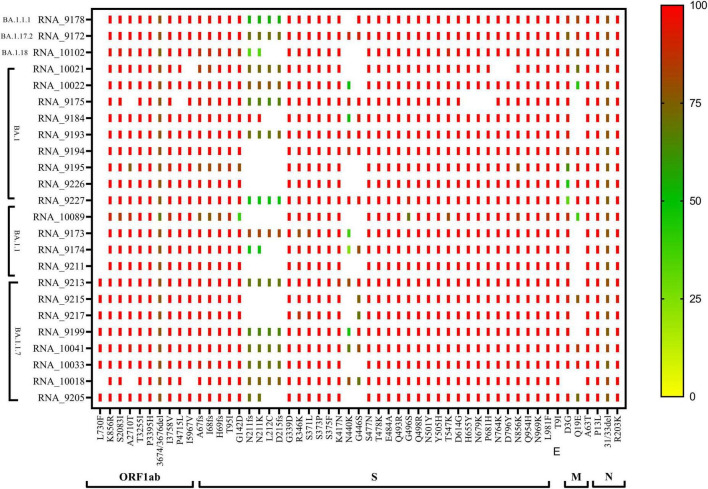
CLC genomics analysis of the mutational changes in the BA.1 and sub-lineages of the Omicron variant. The scale representing the frequency of residue changes from least **(Blue)** to highest **(Red)**. S, spike protein; E, envelope protein; M, membrane protein; N, nucleocapsid protein.

The phylogenetic NJ tree was generated based on the updated pangolin lineage of sequences updated as of 22 April 2022. The Omicron lineage BA.1 and sub-lineages BA.1.1, BA.1.1.7, BA.1.1.1, BA.1.17.2, and BA.1.18 showed prominent signature mutations (Figure 2). Also, ORF1ab showed strong frequencies of signature mutations K856R, S2083I, A2710T, T3255I, P3395H, I3758V, P4715L, and I5967V in all lineages, while at position 3674/3676del showing moderate frequencies, the frequency of mutation L730F was only prominent in BA.1.1.7 lineage and absent in other lineages. Spike gene (S gene) showed the signature mutations A67fs, I68fs, H69fs, T95I, G142D, G339, R346K, S371L, S373P, S375F, K417N, T478K, E484A, Q493R, G496S, Q498R, N501Y, Y505H, D614G, H655Y, N679K, P681H, N764K, D796Y, N856K, Q954H, N969K, and L981F with >90% frequency, but mutations N440K and G446S were found in variable frequency 25–100%, while frameshift N211fs, D215fs, and mutations N211K and L212C were observed with frequency 25–75%. N gene showed signature mutations P13L and R203K with > 90% frequency, while deletion 31/33del with frequency 75–80% (Figure 2).

**FIGURE 3:**
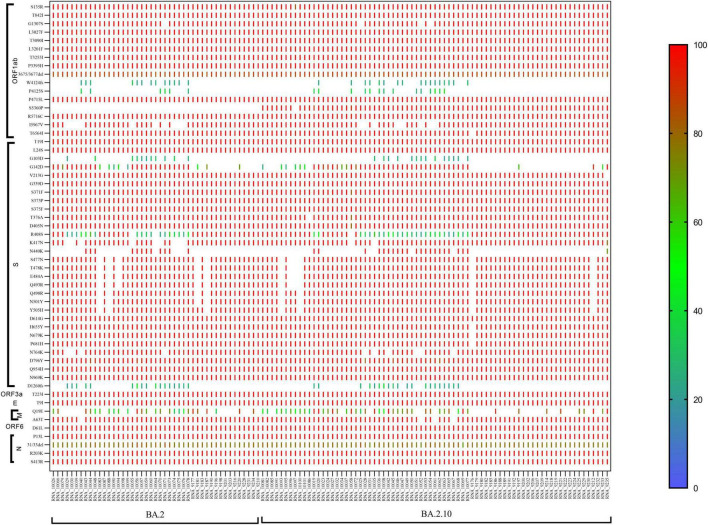
CLC genomics showing the mutational changes in the sub-lineage BA.2 and BA.2.10 of the Omicron variant. The scale representing the frequency of residue changes from least **(Blue)** to highest **(Red)**. S, spike protein; E, envelope protein; M, membrane protein; N, nucleocapsid protein.

The majority of the sequences belonged to Omicron BA.2 (*n* = 45) and sub-lineages BA.2.10 (*n* = 74), BA.2.12 (*n* = 01) pangolin lineage. In BA.2 lineage, the ORF1ab region showed the signature mutations S135R, T842I, G1307S, L3027F, T3090I, L3201F, T3255I, P3395H, P4715L, R5716C, I5967V, I5967V, and T6564I, while moderate frequency showed by 13% sequences at position P4125S and 28% sequences at W4124fs, but there is no mutation observed at S5360P. Spike region (S gene) with 100% prominent signature mutations showed T19I, L24S, V213G, G339D, S371F, S373P, S375F, T376A, D405N, D614G, H655Y, N679K, P681H, D796Y, Q954H, and N969K mutations, but 88% sequences showed mutations S477N, T478K, E484A, and Q493R, 91% sequences showed Q498R, N501Y, and Y505H amino acid mutation, D1260fs showed frameshift in 17 sequences with low frequency 20–60%, while no frameshift was observed in 28 sequences. ORF3a showed signature mutation T223I, whereas the M gene showed frequency variability (30–100%) of signature mutation Q19E. The N gene showed signature mutation P13L, R203K, and S413R, but position 31/33del showed deletion with 80-90% frequency. In the sub-lineage BA.2.10, the signature mutations S135R, T842I, G1307S, L3027F, T3090I, L3201F, T3255I, P3395H, P4715L, R5716C, and T6564I present with 90–100% frequency. Position 3675/3677del showed deletion with 80% frequency. The 22% cases showed frameshift at the W4124fs position, while 18% cases showed P4125S mutation with 20–60% frequency, but signature mutation was observed at S5360P with >90% frequency which was not observed in BA.2 lineage. In spike gene, signature mutations T19I, L24S, V213G, G339D, S371F, S373P, S375F, T376A, D405N, K417N, K477N, T478K, E484A, Q493R, Q498R, N501Y, Y505H, D614G, H655Y, N679K, P681H, D796Y, Q954H, and N969K present in >90% frequency. Only 28% of cases were observed with N440K mutation, while 27% of cases were observed with frameshift at D1260fs with 20–60% frequency. The N gene showed all the signature mutations P13L, R203K, and S413R with >90% frequency and deletion at 31/33del position with 60–80% frequency (Figure 3).

### Residual hotspots of omicron severe acute respiratory syndrome coronavirus 2

Sequence analysis revealed that spike glycoprotein of Omicron BA.1 and sub-lineages harbor a total of 45 amino acid alterations (Table 2). Two minor deletions Δ69 and Δ143–144 in the N-Terminal domain (NTD) and one minuscule insertion at position 214 (ins214EPE) in spike glycoprotein with 15, 18, and 17 mutations occurring in the receptor-binding domain (RBD) of BA.1, BA.1.1, and BA.1.1.7, respectively. Interestingly, two residues S:A67V and S:T95I, within the N-terminal domain (NTD); S:S371L, S:G446S, and S:G496S in the RBD; S:T547K at the C-terminus of the S1 subunit; S:N856K present within the region between Fusion peptide (FP), heptad repeat sequence 1 (HR1), and S:L981F located within the HR1 was identified in Omicron BA.1 and sub-lineages only. The spike protein analysis of Omicron variant BA.2 and sub-lineages has shown 32 residue substitutions and one deletion Δ25–26, with 17 mutations occurring in the RBD. The Omicron BA.2 spike protein lacks Δ69, which is associated with S gene target failure. The Omicron variant has triple spike mutations “H655Y + N679K + P681H” near the S1/S2 junction (residues 681–685) to the furin cleavage site.

**TABLE 2:** Residue substitution in spike protein among various lineages of SARS-CoV-2 virus.

SARS-CoV-2 VOC	No.	Spike amino acid changes in SARS-CoV-2 compared with Wuhan-Hu-1 isolate
Delta B.1.617.2	2	S:T19R, S:T95I, S:L452R, S:T478K, S:D614G, S:P681R, S:A684V, S:V1104L, S:I1114V
Omicron BA.1 and sub-lineages	24	S:A67V, S: H69-, S:G75R*, S:T95I, S: V143-, S:Y144-, S:N165K*, S:ins214EPE, S:G339D, S:R346K*, S:S371L, S:S373P, S:S375F, S:K417N, S:N440K*, S:G446S, S:S477N, S:T478K, S:E484A, S:Q493R, S:G496S, S:Q498R, S:N501Y, S:Y505H, S:T547K, S:D614G, S:F643L*, S:H655Y, S:N679K, S:P681H, S:A684V*, S:N764K, S:D796Y, S:N856K, S:Q954H, S:N969K, S:L981F
Omicron BA.2 and sub-lineages	120	S:T19I, S:L24S, S: P25-, S: P26-, S:G142D, S:V213G, S:G339D, S:S371F, S:S373P, S:S375F, S:T376A, S:D405N, S:K417N, S:S477N, S:T478K, S:E484A, S:Q493R, S:Q498R, S:N501Y, S:Y505H, S:D614G, S:H655Y, S:N679K, S:P681H, S:S704L*, S:N764K, S:D796Y, S:Q954H, S:N969K

*Indicates unique residues within the group of BA.1 and BA.2 sub-lineages.

**FIGURE 4:**
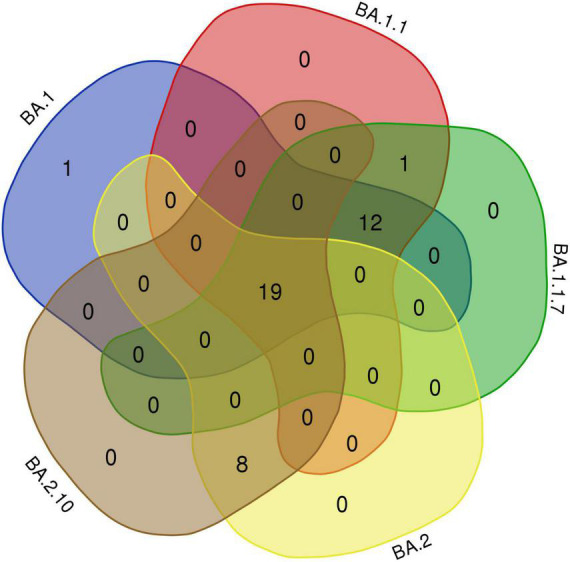
The Venn diagram shows shared mutations in the spike protein mutations of the BA.1, BA.1.1, BA.1.1.7, BA.2, and BA.2.10 lineages in Omicron variants of this study (https://bioinformatics.psb.ugent.be/webtools/Venn/).

Interestingly, 12 amino acids were found common among Omicron variants BA.1, BA.1.1, and BA.1.1.7; a total of 20 amino acids are shared among BA.1, BA.1.1, BA.1.1.7, BA.2, and BA.2.10 strains; and eight residues were common between BA.2 and BA.2.10 (Figure 4 and Table 3). Notably, the S:R346K mutation was exclusively identified in Omicron variants BA.1.1 and BA.1.1.7 only. Moreover, Delta variants differed at positions S:T19R, S:L452R, and S:P681R residues compared with spike glycoprotein of the Omicron variant.

**TABLE 3:** Shared amino acid substitutions in the spike protein of BA.1, BA.1.1, BA.1.1.7, BA.2, and BA.2.10 lineages.

Omicron variant	No.	Shared Spike amino acids between omicron variants
BA.1, BA.1.1, BA.1.1.7, BA.2, BA.2.10	19	S:S373P, S:S375F, S:G339D, S:K417N, S:S477N, S:T478K, S:E484A, S:Q493R, S:Q498R, S:N501Y, S:Y505H, S:N679K, S:P681H, S:D614G, S:H655Y, S:N764K, S:D796Y, S:Q954H, S:N969K
BA.1, BA.1.1, BA.1.1.7	12	S:A67V, S: H69-, S:T95I, S: V143-, S: Y144-, S:ins214EPE, S:S371L, S:G446S, S:G496S, S:T547K, S:N856K, S:L981F
BA.1.1, BA.1.1.7	1	S:R346K
BA.1	1	S:N440K
BA.2 BA.2.10	8	S: P25-, S: P26-, S:T19I, S:V213G, S:G142D, S:S371F, S:T376A, S:D405N,

Furthermore, we analyzed the impact of variation in the RBD region of spike protein among SARS-CoV-2 variants by comparing the sequence conservation among Omicron variants obtained in this study (Figure 5). The results showed that two residue substitutions S371F and T376A, phenylalanine (F) and alanine (A), were found most abundant in the Omicron variant BA.2, which has been replaced completely by leucine (L) and threonine (T), correspondingly, in all the Omicron BA.1 strain, except one sequence (MCL-22-H-3167). An amino acid substitution (N440K) located in the S1 subunit of the spike region, asparagine (46/74), was detected as the most common residue in BA.2.10 variant followed by BA.2 (26/46) and BA.1.1 (4/4), while lysine (K) was the most conserved residue in BA.1 (9/9) variant. In residue substitutions G446S and G496S, glycine was the most common residue with abundance (45/46) and (74/74) in BA.2 and BA.2.10, respectively, while serine (S) was exclusively present at both sites in Omicron variant BA.1 and sub-lineages.

**FIGURE 5:**
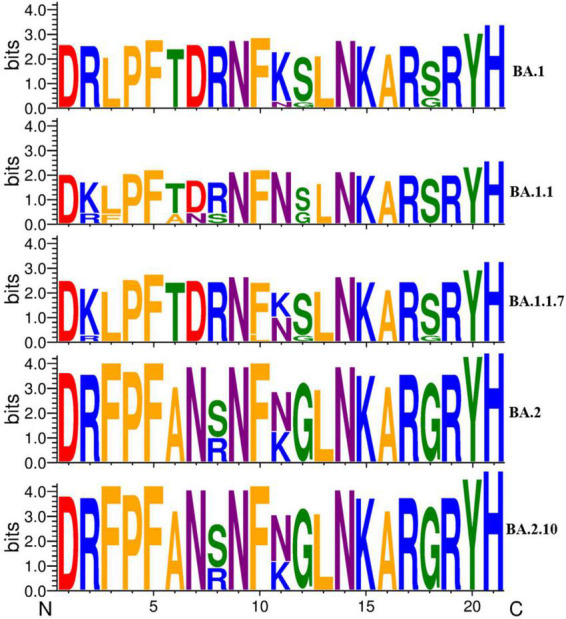
A sequence logo representation of the RBD region of major Omicron variant spike protein in which letter height reflects the likelihood of finding a particular residue in that position. Residues are colored according to hydrophobicity (orange—hydrophobic, blue—basic, red—acidic, purple—neutral, green—polar).

## Discussion

This study reveals the predominance of the Omicron variant in most samples from the eastern part of UP. GRA clade was associated in most cases followed by the GK clade. Omicron variant BA.2 (82.3%) accounted for the most followed by BA.1 (8.8%) and BA.1.1 (7.5%). However, only two samples from asymptomatic cases from the Maharajganj district revealed the presence of circulation of Delta variants (AY.20 and AY.4.2.3). These findings revealed the replacement of the Delta variant which predominated in the second wave with the Omicron variant in the third wave. A previous study from the same center had shown the dominance of the SARS-CoV-2 variant belonging to clades GH, GR, G, S, and O in the first wave followed by G clade variants mostly the Delta and its variant Delta AY.1 and Kappa (17). Neutralization studies have also substantiated the shift of the Omicron variant as a dominant strain replacing the Delta variant (21).

The sociodemographic characteristics specifically regarding the age and gender distribution were similar to those seen in the first wave picture in eastern UP, while the older age group was more affected in the second wave (22). The age distribution was also consistent with the study performed by Raju et al. in Chennai and Garg et al. in Delhi, where the median age was 37 and 35 years, respectively (18, 19). A higher proportion of cases reported in both studies were male. The clinical characteristics showed the comorbidity status (7%) to be much lesser compared with the findings from studies carried out in Chennai (20%) and Delhi (18%). Compared with the symptom status, our study showed a higher symptom rate than the study from Chennai and Delhi (80 vs. 65% and 35%, respectively) (18, 19). Although fever formed the most common symptom, our study reported much higher rates of sore throat, running nose, and body aches. As our study had a smaller sample size and involved confirmed rather than suspected Omicron cases, the discordant findings noted may need further evaluation to draw a more meaningful conclusion.

In this study, the proportion of cases with previous COVID-19 infection was about three times higher than in the Chennai study (16.3 vs. 6%) and similar to the findings of the Delhi study (17%) (18, 19). With respect to travel history, it was lesser than that reported in the Delhi study (31 vs. 39%). Similarly, fully vaccinated individuals having Omicron infection (breakthrough infection) were higher in our study compared with the Chennai study (78 vs. 67%), but lesser than that reported in the Delhi study (88%) (18). The current study noted that breakthrough infection was higher among healthcare workers as reported in previous studies (23, 24). The study further adds to the knowledge showing that the Omicron variant was indeed capable of evading the immunity provided by the vaccine. Furthermore, the current study highlights the median duration from the second dose to Omicron infection to be around 6 months. Raju et al. showed that about three-fourth of the cases had exceeded at least 3 months from the date of the second vaccination (19). This calls for universal administration of third or precautionary doses across all age groups irrespective of comorbidity status.

Sharma et al. conducted a WGS analysis of the SARS-CoV-2-positive cases in Rajasthan obtained from 24 November 2021 to 4 January 2022, and found a predominance of the Delta variant and emergence of the Omicron variant, reflecting the replacement of the Delta variant over the period (20). In the Delhi study which had cases in December 2021, the initial few cases (73.1%) were of BA.1, while 26.8% were of BA.2 sub-lineage (18). Similarly, a study that looked into 59 Omicron cases from various states during the first week of December reported all cases belonging to BA.1 lineage except one case (25). This shift of sub-lineage was evident at the start of the Omicron wave which then took over as seen in our findings in cases seen in January and February 2022 wherein most cases were of BA.2 sub-lineages (82.3%). Among the different lineages of Omicron reported worldwide, BA.1 is the most predominant lineage followed by BA.2. and BA.3 is less reported (26). As there are no reported studies from India, the authors tried to check the most common lineage available in the outbreak information tool and found that BA.2 was the most common lineage reported in India (approximately 55%) as per the data available for the third wave (27).

The emergence of the hypermutated Omicron strain (B.1.1.529) of SARS-CoV-2 has caused serious concern about antibody response due to the number of mutations in spike proteins during the COVID-19 pandemic. The highly mutated Omicron variant is found more contagious but less deadly than the Delta variant of SARS-CoV-2. Molecular sequencing evidence provides information on how a mutation in the spike protein leads to reduced neutralization activity of monoclonal antibodies (mAbs) (28, 29). Omicron (21L, BA.2) spike glycoprotein had the absence of deletion at site 69, which is not identified by SGTF. The deletion at site 69 was also identified in the Alpha and Eta (B.1.525) variants and is associated with enhanced cell-cell fusion and splicing spike into virions (30). Our study found that the S:R346K mutation, which was only present in Omicron variant BA.1.1 recently reported by VanBlargan et al., may be critically important in neutralizing mAbs, suggesting that mutations at these sites may play a crucial role in altering protein structure (31). The amino acid substitutions at positions 1111–1130 in SARS-CoV-2, located upstream of heptad repeat 2 (HR2) segments in the S2 subunit, are associated with viral escape from neutralizing antibodies (32).

The presence of N440K, T478K, and N501Y mutations is very crucial for RBD and the human ACE2 receptor interactions (33), Omicron has a significant transmission potential than the original SARS-CoV-2, which may affect COVID-19 infectivity, neutralizing antibody escapes, and vaccination breakthrough cases (34, 35). Due to its RBD mutations K417N, E484A, and Y505H, Omicron has a higher potential to affect the interaction between 132 antibodies with spike protein, suggesting that it has a higher immune escape ability than the Delta variant (36). Additionally, the D614G substitution located in the S protein is conserved in all variants of Omicron, which increases the infectivity of the SARS-CoV-2 virus (37). Omicron shares the T418K substitution with Delta variants of this study. The triple spike mutations “H655Y + N679K + P681H” lead to enhance replication ability and infectivity of the virus (38). Mechanistically, the elimination of proline residue at spike position 681 results in the inhibition of the glycosylation site found in spike wild-type SARS-CoV-2 Wuhan strain, which could aid transmission (39). Mutation L981F may enhance the interaction affinity between HR1 and HR2, leading to enhanced membrane fusion and infectivity (40). A recent study demonstrated that Omicron BA.2 variant is 30% and 17-fold more capable than BA.1 and Delta, respectively, to escape current vaccines (41). Recently, a case of a breakthrough infection was reported with Delta derivative (AY.112) despite the hybrid immunity followed by reinfection with Omicron derivative BA.2 post-breakthrough infection (42). Recently, a study by Wang et al. found that Del69-70, L452R, F486V, and R493Q mutation sites in the spike protein of the Omicron variant accelerated the spread of the virus and enhanced pathogenicity (43). This calls for further explorations on immunogenicity, vaccine effectiveness, and scheduling of available vaccines for all age groups.

### Strengths and limitations

This study has WGS for a larger sample set giving a true picture of circulating strains with regards to the Omicron variant in eastern UP, India. We could retrieve sequences from all samples with a genome coverage of ≥98% as we used samples having a high viral load. The current study generated data that contributed to adding evidence of vaccine immune evasion of the Omicron variant by determining the breakthrough infections among completely vaccinated individuals and advocating the importance of booster or precautionary doses among all age groups.

Despite all our efforts made to contact all the cases, we could not contact around one-third of the cases to document a complete clinical picture; still, the sample describing a clinical picture is the largest compared with the published studies from India discussing confirmed Omicron cases. With stricter Ct value cut-offs, we were not able to distribute the samples in such a way that could have given clear monitoring across Omicron lineages over days to weeks in the considered 2 months in our study which could have added better insights.

## Conclusion

This study reveals the predominance of the Omicron variant in eastern UP during the third wave of this pandemic. BA.2 lineages of the Omicron predominated replacing the BA.1 lineage over a period. Most of the cases had a breakthrough infection following the recommended two doses of vaccine; however, a subset of cases developing an infection following the precautionary dose was also noted. The current study also had about eight in 10 cases being symptomatic but neither hospitalized nor mortality indicating the less virulence nature of the Omicron variant compared with the much fatal Delta strain. There is a need to further explore the immune evasion properties of the Omicron variant which may be essential in planning for vaccine advocacy in the future in India.

## Data availability statement

The data presented in the study are deposited in the GISAID database, accession numbers: EPI_ISL_12157232-EPI_ISL_12157237, EPI_ISL_12155377-EPI_ISL_12155438, and EPI_ISL_12155440-EPI_ISL_1215551. All data has been released and is available to the public.

## Ethics statement

The studies involving human participants were reviewed and approved by the Institutional Ethics Committee, Indian Council of Medical Research–Regional Medical Research Centre (ICMR–RMRC) Gorakhpur. Written informed consent for participation was not provided by the participants’ legal guardians/next of kin because: Informed verbal telephonic consent was taken prior to the start of the telephonic survey.

## Author contributions

KZ, SM, MR, and RK: conceptualization. AS, SM, AbK, MR, SY, TM, SB, NK, SP, AS, AK, NaK, VK, KK, RSS, MK, ST, and AV: data curation. KZ, AS, SM, AbK, MR, RRS, TM, MD, YJ, PG, and PY: formal analysis. PY and RK: funding acquisition. KZ, AS, SM, AbK, SY, AP, GD, HD, RS, SB, NK, SP, AsK, AS, AK, NaK, VK, KK, RSS, MK, ST, and AV: investigation. KZ, AS, SM, AbK, MR, RRS, SY, TM, AP, GD, HD, RS, AsK, PY, and RK: methodology. KZ, AP, PY, and RK: project administration and resources. KZ, AS, SM, AbK, MR, RRS, SY, TM, GD, NK, SP, MD, YJ, PG, AK, NaK, VK, and KK: software. KZ, GD, RS, PY, and RK: supervision. AS, SM, AbK, MR, SY, TM, MD, YJ, and PG: validation. AS, SM, AbK, RRS, HD, SB, SP, AsK, MD, YJ, AS, PG, AK, RSS, MK, ST, and AV: visualization. KZ, AS, SM, AbK, MR, and RRS: writing – original draft. KZ, MR, PY, and RK: writing – review and editing. All authors approved the final manuscript.

## Footnotes

1
https://up.gov.in/en

2
cvstatus.icmr.gov.in

3
https://www.epicov.org/epi3/frontend#5ff51a

4
https://pangolin.cog-uk.io/

## References

[1] Wu F Zhao S Yu B Chen YM Wang W Song ZG Author correction: a new coronavirus associated with human respiratory disease in China. *Nature.* (2020) 580:E7. 10.1038/s41586-020-2202-3 32296181 PMC7608129

[2] WHO. *Coronavirus (COVID-19) Dashboard.* Geneva: World Health Organization (2022).

[3] MoHFW. *COVID-19 Statewise Status.* New Delhi: Ministry of Health and Family Welfare, Government of India (2022).

[4] Vaidyanathan G. Coronavirus variants are spreading in India - what scientists know so far. *Nature.* (2021) 593:321–2. 10.1038/d41586-021-01274-7 33976409

[5] Kirola L. Genetic emergence of B.1.617.2 in COVID-19. *New Microbes New Infect.* (2021) 43:100929. 10.1016/j.nmni.2021.100929 34336227 PMC8302888

[6] World Health Organization. *Classification of Omicron (B.1.1.529): SARS-CoV-2 Variant of Concern.* Geneva: World Health Organization (2021).

[7] Sarkar A Chakrabarti AK Dutta S. Covid-19 infection in India: a comparative analysis of the second wave with the first wave. *Pathogens.* (2021) 10:1222. 10.3390/pathogens10091222 34578254 PMC8469101

[8] Gupta N Kaur H Yadav PD Mukhopadhyay L Sahay RR Kumar A Clinical characterization and genomic analysis of samples from COVID-19 breakthrough infections during the second wave among the various states of India. *Viruses.* (2021) 13:1782. 10.3390/v13091782 34578363 PMC8472862

[9] Sarkar R Saha R Mallick P Sharma R Kaur A Dutta S Emergence of a novel SARS-CoV-2 Pango lineage B.1.1.526 in West Bengal, India. *J Infect Public Health.* (2022) 15:42–50. 10.1016/j.jiph.2021.11.020 34896696 PMC8642833

[10] Tegally H Moir M Everatt J Giovanetti M Scheepers C Wilkinson E Emergence of SARS-CoV-2 Omicron lineages BA.4 and BA.5 in South Africa. *Nat Med.* (2022) 28:1785–90. 35760080 10.1038/s41591-022-01911-2 PMC9499863

[11] Mohapatra RK Sarangi AK Kandi V Azam M Tiwari R Dhama K. Omicron (B.1.1.529 variant of SARS-CoV-2); an emerging threat: current global scenario. *J Med Virol.* (2022) 94:1780–3. 10.1002/jmv.27561 34964506 PMC9015454

[12] Kim S Liu Y Ziarnik M Cao Y Zhang XF Im W. Binding of human ACE2 and RBD of Omicron enhanced by unique interaction patterns among SARS-CoV-2 variants of concern. *bioRxiv* [Preprint]. (2022): 10.1101/2022.01.24.477633 36398990 PMC9825653

[13] Kumar S Thambiraja TS Karuppanan K Subramaniam G. Omicron and delta variant of SARS-CoV-2: a comparative computational study of spike protein. *J Med Virol.* (2022) 94:1641–9. 10.1002/jmv.27526 34914115

[14] Fantini J Yahi N Colson P Chahinian H La Scola B Raoult D. The puzzling mutational landscape of the SARS-2-variant Omicron. *J Med Virol.* (2022) 94:2019–25. 10.1002/jmv.27577 34997962 PMC9015223

[15] Meng B Abdullahi A Ferreira I Goonawardane N Saito A Kimura I Altered TMPRSS2 usage by SARS-CoV-2 Omicron impacts infectivity and fusogenicity. *Nature.* (2022) 603:706–14. 35104837 10.1038/s41586-022-04474-x PMC8942856

[16] Bruel T Hadjadj J Maes P Planas D Seve A Staropoli I Serum neutralization of SARS-CoV-2 Omicron sublineages BA.1 and BA.2 in patients receiving monoclonal antibodies. *Nat Med.* (2022) 28:1297–302. 10.1038/s41591-022-01792-5 35322239

[17] Deval H Nyayanit DA Mishra SK Yadav PD Zaman K Shankar P Genome sequencing reveals a mixed picture of SARS-CoV-2 variant of concern circulation in Eastern Uttar Pradesh, India. *Front Med (Lausanne).* (2021) 8:781287. 10.3389/fmed.2021.781287 35071267 PMC8777020

[18] Garg R Gautam P Suroliya V Agarwal R Bhugra A Kaur US Evidence of early community transmission of Omicron (B1.1.529) in Delhi– a city with very high seropositivity and past-exposure. *Travel Med Infect Dis.* (2022) 46:102276. 10.1016/j.tmaid.2022.102276 35181557 PMC9759830

[19] Raju MK Thangaraj JWV Selvavinayagam TS Somasundaram A Parthipan K Sivadoss R Clinical profile of patients infected with suspected SARS-CoV-2 Omicron variant of concern, Tamil Nadu, India, December 2021-January 2022. *Indian J Med Res.* (2022) 155:165–70. 10.4103/ijmr.ijmr_312_22 35417991 PMC9552397

[20] Sharma RP Gautam S Sharma P Singh R Sharma H Parsoya D Clinico epidemiological profile of Omicron variant of SARS CoV2 in Rajasthan. *medRxiv* [Preprint]. (2022). 10.1101/2022.02.11.22270698

[21] Yadav PD Sapkal GN Sahay RR Potdar VA Deshpande GR Patil DY Substantial immune response in Omicron infected breakthrough and unvaccinated individuals against SARS-CoV-2 variants of concern. *J Infect.* (2022) 84:e80–1. 10.1016/j.jinf.2022.02.005 35157945 PMC8837481

[22] Reddy MM Zaman K Mishra SK Yadav P Kant R. Differences in age distribution in first and second waves of COVID-19 in eastern Uttar Pradesh, India. *Diabetes Metab Syndr.* (2021) 15:102327. 10.1016/j.dsx.2021.102327 34731821 PMC8556065

[23] Kumar N Misra BR Reddy MM Deval H Zaman K Kant R. COVID-19 transmission among vaccinated laboratory workers during the second wave in eastern Uttar Pradesh, India. *J Med Virol.* (2022) 94:3512–4. 10.1002/jmv.27788 35434827 PMC9088332

[24] Kaur U Bala S Ojha B Jaiswal S Kansal S Chakrabarti SS. Occurrence of COVID-19 in priority groups receiving ChAdOx1 nCoV-19 coronavirus vaccine (recombinant): a preliminary analysis from north India. *Res Squ.* (2021) 94:407–12. 10.21203/rs.3.rs-772465/v1 PMC8662184 34491572

[25] Potdar VA Yadav PD Lole K Cherian S Shastri J Malhotra B Detection of the Omicron variant in international travellers and their family contacts in India. *medRxiv* [Preprint]. (2021). 10.1101/2021.12.27.21268429

[26] Desingu PA Nagarajan K Dhama K. Emergence of Omicron third lineage BA.3 and its importance. *J Med Virol.* (2022) 94:1808–10. 10.1002/jmv.27601 35043399 PMC9015590

[27] Gangavarapu K Latif AA Mullen J Alkuzweny M Hufbauer E Tsueng G Outbreak info genomic reports: scalable and dynamic surveillance of SARS-CoV-2 variants and mutations. *Res Sq.* [Preprint]. (2022). 10.21203/rs.3.rs-1723829/v1 36823332 PMC10399614

[28] Li Q Nie J Wu J Zhang L Ding R Wang H SARS-CoV-2 501Y.V2 variants lack higher infectivity but do have immune escape. *Cell.* (2021) 184:2362.e–71.e. 10.1016/j.cell.2021.02.042 33735608 PMC7901273

[29] Planas D Bruel T Grzelak L Guivel-Benhassine F Staropoli I Porrot F Sensitivity of infectious SARS-CoV-2 B.1.1.7 and B.1.351 variants to neutralizing antibodies. *Nat Med.* (2021) 27:917–24. 10.1038/s41591-021-01318-5 33772244

[30] Meng B Kemp SA Papa G Datir R Ferreira I Marelli S Recurrent emergence of SARS-CoV-2 spike deletion H69/V70 and its role in the Alpha variant B.1.1.7. *Cell Rep.* (2021) 35:109292. 10.1016/j.celrep.2021.109292 PMC8185188 34166617

[31] VanBlargan LA Errico JM Halfmann PJ Zost SJ Crowe JE Jr. Purcell LA An infectious SARS-CoV-2 B.1.1.529 Omicron virus escapes neutralization by therapeutic monoclonal antibodies. *Nat Med.* (2022) 28:490–5. 10.1038/s41591-021-01678-y 35046573 PMC8767531

[32] Ng OW Keng CT Leung CS Peiris JS Poon LL Tan YJ. Substitution at aspartic acid 1128 in the SARS coronavirus spike glycoprotein mediates escape from a S2 domain-targeting neutralizing monoclonal antibody. *PLoS One.* (2014) 9:e102415. 10.1371/journal.pone.0102415 25019613 PMC4097068

[33] Starr TN Greaney AJ Hilton SK Ellis D Crawford KHD Dingens AS Deep mutational scanning of SARS-CoV-2 receptor binding domain reveals constraints on folding and ACE2 binding. *Cell.* (2020) 182:1295.e–310.e. 10.1016/j.cell.2020.08.012 32841599 PMC7418704

[34] Ali F Kasry A Amin M. The new SARS-CoV-2 strain shows a stronger binding affinity to ACE2 due to N501Y mutant. *Med Drug Discov.* (2021) 10:100086. 10.1016/j.medidd.2021.100086 33681755 PMC7923861

[35] Chen J Wang R Gilby NB Wei GW. Omicron variant (B.1.1.529): infectivity, vaccine breakthrough, and antibody resistance. *J Chem Inf Model.* (2022) 62:412–22. 10.1021/acs.jcim.1c01451 34989238 PMC8751645

[36] Saxena SK Kumar S Ansari S Paweska JT Maurya VK Tripathi AK Transmission dynamics and mutational prevalence of the novel severe acute respiratory syndrome coronavirus-2 Omicron variant of concern. *J Med Virol.* (2022) 94:2160–6. 10.1002/jmv.27611 35050521 PMC9015611

[37] Korber B Fischer WM Gnanakaran S Yoon H Theiler J Abfalterer W Tracking changes in SARS-CoV-2 spike: evidence that D614G increases infectivity of the COVID-19 virus. *Cell.* (2020) 182:812–27.e. 10.1016/j.cell.2020.06.043 32697968 PMC7332439

[38] Mertens J Coppens J Loens K Le Mercier M Xavier BB Lammens C Monitoring the SARS-CoV-2 pandemic: screening algorithm with single nucleotide polymorphism detection for the rapid identification of established and emerging variants. *Clin Microbiol Infect.* (2022) 28:124–9. 10.1016/j.cmi.2021.09.007 34537361 PMC8444474

[39] Planas D Saunders N Maes P Guivel-Benhassine F Planchais C Buchrieser J Considerable escape of SARS-CoV-2 Omicron to antibody neutralization. *Nature.* (2022) 602:671–5. 10.1038/s41586-021-04389-z 35016199

[40] Sarkar R Lo M Saha R Dutta S Chawla-Sarkar M. S glycoprotein diversity of the Omicron variant. *medRxiv* [Preprint]. (2021). 10.1101/2021.12.04.21267284

[41] Chen J Wei G-W. Omicron BA.2 (B.1.1.529.2): high potential to becoming the next dominating variant. *arXiv* [Preprint]. (2022). arXiv:2202.05031v05031, 10.21203/rs.3.rs-1362445/v1 35233567 PMC8887081

[42] Pandit P Bhatt P Sahay RR Joshi Y Patil DY Yadav PD. A case of breakthrough infection with SARS-CoV-2 Delta derivative and reinfection with Omicron variant in a fully vaccinated health care professional. *J Infect.* (2022) 85:e15–7. 10.1016/j.jinf.2022.04.023 35447232 PMC9014656

[43] Wang Q Guo Y Iketani S Nair MS Li Z Mohri H Antibody evasion by SARS-CoV-2 Omicron subvariants BA.2.12.1, BA.4 and BA.5. *Nature.* (2022) 608:603–8. 10.1038/s41586-022-05053-w 35790190 PMC9385487

