# Supplementary material for: Omicron BA.2 lineage predominance in severe acute respiratory syndrome coronavirus 2 positive cases during the third wave in North India
Source: Front Med (Lausanne). 2022 Nov 2;9:955930. doi: 10.3389/fmed.2022.955930 (PMC9666497; doi:10.3389/fmed.2022.955930)
Supplement: Supplementary file 1 [file Table_1.DOCX]

**Supplementary Table 1: Districtwise distribution of socio-demographic, clinical and genomic characteristics of sequenced SARS-CoV-2 positive cases during third wave (01 January to 24 February 2022) in eastern Uttar Pradesh (N = 147)**

| **Parameter** | **Deoria (n= 43)** | **Gorakhpur(n=43)** | **Maharajganj(n=61)** |
| --- | --- | --- | --- |
| **Age in years (Mean/IQR)** | 32.8/15 | 30.7/14 | 37.1/21 |
| **Gender (M/F)** | 32/11 | 33/10 | 43/18 |
| **Healthcare worker** | 5 | 28 | 1 |
| **SARS-CoV-2 variants among genome retrieved (n= 146)** | | | |
| BA.1 | 1 | 1 | 7 |
| BA.1.1 | 3 | 1 | 0 |
| BA.1.1.1 | 1 | 0 | 0 |
| BA.1.1.7 | 5 | 1 | 2 |
| BA.1.17.2 | 1 | 0 | 0 |
| BA.1.18 | 0 | 1 | 0 |
| BA.2 | 13 | 16 | 17 |
| BA.2.10 | 19 | 22 | 33 |
| BA.2.12 | 0 | 1 | 0 |
| B.1.617.2 | 0 | 0 | 2 |
| **Type of vaccine (n = 98)** |  |  |  |
| BBIBP-CorV | 3 | 0 | 0 |
| ChAdOx1 nCoV-19 | 26 | 28 | 24 |
| BBV152 | 1 | 0 | 3 |
| Not vaccinated | 4 | 6 | 3 |
| **Vaccination status (n = 98)** |  |  |  |
| 1^st^ +2^nd^ + Precautionary dose | 5 | 1 | 3 |
| 1^st^ and 2^nd^ dose | 20 | 26 | 24 |
| Only 1^st^ dose | 5 | 1 | 0 |
| **Travel history** | | | |
| Domestic | 6 | 9 | 8 |
| International | 5 | 0 | 2 |
| **Symptom status (n = 98)** | | | |
| Symptomatic | 25 | 30 | 24 |
| Asymptomatic | 9 | 4 | 6 |
| **Sign and Symptoms** | | | |
| Fever | 22 | 29 | 19 |
| Cough | 22 | 26 | 21 |
| Running nose |  |  | 2 |
| Sore throat | 17 | 22 | 17 |
| Body ache | 10 | 16 | 8 |
| Headache | 1 | 13 | 2 |
| Breathing shortness | 3 | 3 | 3 |
| Loss of taste | 2 | 3 | 1 |
| **Co-morbidity** | | | |
| Diabetes mellitus | 1 | 2 | 1 |
| Hypertension | 0 | 0 | 1 |
| Tuberculosis | 0 | 0 | 1 |
| bronchial asthma | 0 | 0 | 1 |
| **SARS-CoV-2 re-infection** | 3 | 10 | 3 |
