# Supplementary material for: Omicron BA.2 lineage predominance in severe acute respiratory syndrome coronavirus 2 positive cases during the third wave in North India
Source: Front Med (Lausanne). 2022 Nov 2;9:955930. doi: 10.3389/fmed.2022.955930 (PMC9666497; doi:10.3389/fmed.2022.955930)
Supplement: Supplementary file 2 [file Table_2.DOCX]

**Supplementary Table 2: Summary of Whole-genome sequencing of positive samples infected with SARS-CoV-2 virus.**

| **S.No.** | **GISAID ID** | **Age** | **Gender** | **Avg. Ct value E gene** | **Travelling History** | **Total Read** | **Relevant read** | **Length Recovered** | **% Genome recovered** | **Circulating Clade** | **Pangolin Lineage as on 22.04.2022 used for tree** |
| --- | --- | --- | --- | --- | --- | --- | --- | --- | --- | --- | --- |
| 1 | EPI_ISL_12155457 | 44 | male | 16.76 | Dubai | 8,92,504 | 7,70,486 | 29814 | 99.70 | GRA | BA.1.17.2 |
| 2 | EPI_ISL_12155458 | 23 | male | 25.3 | Dubai | 9,92,448 | 9,59,329 | 29601 | 98.99 | GRA | BA.1.1 |
| 3 | EPI_ISL_12155459 | 33 | male | 19.22 | Dubai | 11,17,408 | 10,47,885 | 29804 | 99.67 | GRA | BA.1.1 |
| 4 | EPI_ISL_12155460 | 30 | female | 17.2 | Dubai | 10,29,518 | 9,70,457 | 29425 | 98.40 | GRA | BA.1 |
| 5 | EPI_ISL_12157235 | 31 | male | 22.42 | nd | 10,44,166 | 9,12,409 | 28723 | 96.05 | GRA | BA.2.10 |
| 6 | EPI_ISL_12155461 | 30 | male | 19.05 | Delhi | 9,12,409 | 10,70,942 | 29721 | 99.39 | GRA | BA.2 |
| 7 | EPI_ISL_12155462 | 36 | male | 23.49 | nd | 9,18,302 | 7,94,403 | 29560 | 98.85 | GRA | BA.1.1.1 |
| 8 | EPI_ISL_12155463 | 18 | female | 22.5 | na | 10,47,112 | 9,98,725 | 29802 | 99.66 | GRA | BA.2.10 |
| 9 | EPI_ISL_12155464 | 29 | male | 23.69 | na | 9,99,046 | 9,60,495 | 29800 | 99.66 | GRA | BA.2.10 |
| 10 | EPI_ISL_12155465 | 58 | male | 22.91 | nd | 1,23,082 | 1,12,694 | 29674 | 99.23 | GRA | BA.2 |
| 11 | EPI_ISL_12155466 | 42 | male | 17.71 | Chattisgarh | 11,29,658 | 10,95,548 | 29807 | 99.68 | GRA | BA.2.10 |
| 12 | EPI_ISL_12157236 | 25 | male | 18.09 | na | 9,25,534 | 8,51,165 | 29813 | 99.70 | GRA | BA.2 |
| 13 | EPI_ISL_12155467 | 45 | male | 26.57 | Mathura | 9,74,250 | 9,32,640 | 29699 | 99.32 | GRA | BA.1 |
| 14 | EPI_ISL_12155468 | 38 | male | 17.96 | Patna | 12,03,206 | 11,27,611 | 29803 | 99.67 | GRA | BA.2.10 |
| 15 | EPI_ISL_12155469 | 37 | male | 23.78 | Gorakhpur | 10,26,566 | 8,16,922 | 29506 | 98.67 | GRA | BA.2.10 |
| 16 | EPI_ISL_12155470 | 10 | male | 29.85 | Mathura | 10,35,614 | 9,07,076 | 29251 | 97.82 | GRA | BA.2 |
| 17 | EPI_ISL_12155471 | 48 | male | 19.35 | Delhi | 8,58,404 | 8,14,221 | 29799 | 99.65 | GRA | BA.2.10 |
| 18 | EPI_ISL_12155472 | 32 | male | 19.05 | nd | 7,46,616 | 7,26,300 | 29804 | 99.67 | GRA | BA.2.10 |
| 19 | EPI_ISL_12155473 | 28 | male | 15.65 | Dubai | 11,75,586 | 10,85,734 | 29843 | 99.80 | GRA | BA.2 |
| 20 | EPI_ISL_12155474 | 34 | female | 23.12 | Delhi | 11,64,388 | 9,95,191 | 29719 | 99.38 | GRA | BA.2.10 |
| 21 | EPI_ISL_12155475 | 1 | male | 21.02 | Delhi | 10,26,482 | 7,99,519 | 29799 | 99.65 | GRA | BA.2.10 |
| 22 | EPI_ISL_12155476 | 32 | male | 17.95 | Mumbai | 10,50,372 | 9,89,277 | 29813 | 99.70 | GRA | BA.1 |
| 23 | EPI_ISL_12155477 | 52 | male | 20.74 | nd | 8,36,296 | 5,41,687 | 29380 | 98.25 | GRA | BA.1 |
| 24 | EPI_ISL_12157237 | 27 | male | 27.44 | nd | 9,41,826 | 5,11,249 | 29461 | 98.52 | GRA | BA.1 |
| 25 | EPI_ISL_12155478 | 40 | male | 15.44 | na | 10,26,826 | 9,47,868 | 29805 | 99.67 | GRA | BA.2 |
| 26 | EPI_ISL_12155479 | 38 | female | 24.88 | na | 9,44,194 | 6,70,208 | 29667 | 99.21 | GRA | BA.2.10 |
| 27 | EPI_ISL_12155480 | 30 | female | 23.22 | nd | 10,26,158 | 9,55,858 | 29785 | 99.61 | GRA | BA.2 |
| 28 | EPI_ISL_12155481 | 31 | female | 22.8 | na | 10,75,340 | 10,39,258 | 29813 | 99.70 | GRA | BA.1.1.7 |
| 29 | EPI_ISL_12155482 | 27 | male | 16.49 | Kathmandu | 9,55,196 | 8,95,644 | 29799 | 99.65 | GRA | BA.2.10 |
| 30 | EPI_ISL_12155483 | 42 | male | 23.51 | nd | 11,21,210 | 10,35,545 | 29763 | 99.53 | GRA | BA.2 |
| 31 | EPI_ISL_12155484 | 36 | Female | 23.77 | Delhi | 10,46,178 | 9,36,468 | 29622 | 99.06 | GRA | BA.2.10 |
| 32 | EPI_ISL_12155485 | 35 | Male | 19.82 | nd | 11,69,708 | 9,85,169 | 29799 | 99.65 | GRA | BA.2 |
| 33 | EPI_ISL_12155486 | 47 | male | 25.33 | nd | 7,82,388 | 7,09,966 | 29552 | 98.83 | GK | B.1.617.2 |
| 34 | EPI_ISL_12155487 | 75 | female | 27.42 | nd | 8,95,938 | 6,95,112 | 29310 | 98.02 | GRA | BA.1.1.7 |
| 35 | EPI_ISL_12155488 | 27 | female | 15.25 | nd | 11,23,018 | 10,22,456 | 29802 | 99.66 | GRA | BA.2.10 |
| 36 | EPI_ISL_12155489 | 65 | male | 18.94 | nd | 11,76,048 | 11,25,206 | 29802 | 99.66 | GRA | BA.2.10 |
| 37 | EPI_ISL_12155490 | 30 | male | 26.05 | nd | 8,86,166 | 8,46,675 | 29778 | 99.58 | GK | B.1.617.2 |
| 38 | EPI_ISL_12155491 | 23 | male | 21.72 | Manali | 12,90,148 | 11,96,225 | 29802 | 99.66 | GRA | BA.2.10 |
| 39 | EPI_ISL_12155492 | 31 | male | 19.58 | Hyderabad | 12,17,402 | 11,34,658 | 29804 | 99.67 | GRA | BA.2 |
| 40 | EPI_ISL_12155493 | 60 | male | 23.93 | na | 11,93,426 | 11,46,505 | 29613 | 99.03 | GRA | BA.1.1 |
| 41 | EPI_ISL_12155494 | 16 | female | 28.09 | Gorakhpur | 8,83,180 | 5,87,134 | 29200 | 97.65 | GRA | BA.2.10 |
| 42 | EPI_ISL_12155495 | 40 | male | 16.4 | na | 8,16,294 | 7,86,359 | 29811 | 99.69 | GRA | BA.1.1.7 |
| 43 | EPI_ISL_12155496 | 21 | male | 24.18 | nd | 9,48,700 | 8,54,313 | 29722 | 99.39 | GRA | BA.2.10 |
| 44 | EPI_ISL_12155497 | 29 | male | 24.58 | nd | 10,76,182 | 10,51,357 | 29822 | 99.73 | GRA | BA.1.1.7 |
| 45 | EPI_ISL_12155498 | 28 | male | 22.39 | nd | 10,65,842 | 10,37,899 | 29719 | 99.38 | GRA | BA.2 |
| 46 | EPI_ISL_12155499 | 17 | male | 23.59 | nd | 11,18,654 | 10,11,928 | 29787 | 99.61 | GRA | BA.1.1.7 |
| 47 | EPI_ISL_12155500 | 38 | male | 21.86 | na | 8,58,150 | 8,01,902 | 29805 | 99.67 | GRA | BA.2.10 |
| 48 | EPI_ISL_12155501 | 43 | male | 22.23 | na | 12,17,624 | 11,78,306 | 29802 | 99.66 | GRA | BA.2.10 |
| 49 | EPI_ISL_12155502 | 32 | male | 25.23 | Pune | 9,33,944 | 7,94,209 | 29716 | 99.37 | GRA | BA.2 |
| 50 | EPI_ISL_12155503 | 50 | male | 23.01 | nd | 9,73,264 | 8,55,920 | 29700 | 99.32 | GRA | BA.2.10 |
| 51 | EPI_ISL_12155504 | 26 | female | 16.03 | nd | 9,21,420 | 7,73,768 | 29796 | 99.64 | GRA | BA.2.10 |
| 52 | EPI_ISL_12155505 | 38 | male | 16.44 | nd | 9,60,492 | 9,22,946 | 29803 | 99.67 | GRA | BA.2.10 |
| 53 | EPI_ISL_12155506 | 25 | female | 17.42 | nd | 9,11,018 | 8,61,139 | 29806 | 99.68 | GRA | BA.2.10 |
| 54 | EPI_ISL_12155507 | 45 | female | 21.35 | nd | 11,51,624 | 10,09,955 | 29671 | 99.22 | GRA | BA.2.10 |
| 55 | EPI_ISL_12155508 | 66 | female | 21.96 | nd | 10,97,150 | 10,42,504 | 29700 | 99.32 | GRA | BA.1 |
| 56 | EPI_ISL_12155509 | 60 | female | 16.42 | nd | 11,64,884 | 10,83,798 | 29814 | 99.70 | GRA | BA.1 |
| 57 | EPI_ISL_12155510 | 46 | male | 21.91 | Mumbai | 12,12,796 | 11,75,935 | 29797 | 99.65 | GRA | BA.2 |
| 58 | EPI_ISL_12155511 | 52 | male | 25.03 | nd | 9,54,470 | 8,49,140 | 29694 | 99.30 | GRA | BA.2.10 |
| 59 | EPI_ISL_12155512 | 40 | male | 18.61 | nd | 7,38,884 | 6,37,715 | 29801 | 99.66 | GRA | BA.2.10 |
| 60 | EPI_ISL_12155513 | 28 | male | 25.13 | na | 10,49,336 | 10,16,142 | 29662 | 99.19 | GRA | BA.2 |
| 61 | EPI_ISL_12155514 | 51 | male | 21.08 | na | 11,08,788 | 10,67,474 | 29802 | 99.66 | GRA | BA.2.10 |
| 62 | EPI_ISL_12155515 | 53 | male | 26.71 | nd | 9,37,156 | 8,81,527 | 29491 | 98.62 | GRA | BA.2.10 |
| 63 | EPI_ISL_12155516 | 13 | female | 23.53 | na | 6,17,864 | 5,49,339 | 29368 | 98.21 | GRA | BA.2 |
| 64 | EPI_ISL_12155517 | 42 | male | 28.59 | nd | 6,19,344 | 5,78,263 | 29660 | 99.19 | GRA | BA.2.10 |
| 65 | EPI_ISL_12155377 | 40 | Male | 19.29 | na | 52,182 | 51,384 | 29492 | 98.63 | GRA | BA.1.1.7 |
| 66 | EPI_ISL_12155378 | 23 | Female | 17.39 | na | 1,84,604 | 1,81,350 | 29728 | 99.41 | GRA | BA.2.10 |
| 67 | EPI_ISL_12155379 | 20 | Male | 15.15 | na | 1,76,400 | 1,72,616 | 29607 | 99.01 | GRA | BA.2.10 |
| 68 | EPI_ISL_12155380 | 25 | Female | 24.33 | nd | 2,39,754 | 2,31,844 | 29329 | 98.08 | GRA | BA.1 |
| 69 | EPI_ISL_12155381 | 29 | Male | 23.6 | nd | 5,36,012 | 5,19,547 | 29793 | 99.63 | GRA | BA.1 |
| 70 | EPI_ISL_12155382 | 48 | Female | 23.57 | nd | 5,56,792 | 5,11,208 | 29764 | 99.54 | GRA | BA.2.10 |
| 71 | EPI_ISL_12155383 | 60 | Male | 26.26 | nd | 5,01,690 | 4,62,150 | 29714 | 99.37 | GRA | BA.2.10 |
| 72 | EPI_ISL_12155384 | 50 | Male | 25.1 | na | 2,57,738 | 2,48,806 | 29403 | 98.33 | GRA | BA.2.10 |
| 73 | EPI_ISL_12155385 | 35 | Male | 26.8 | Nepal | 4,77,804 | 4,57,589 | 29761 | 99.53 | GRA | BA.2 |
| 74 | EPI_ISL_12155386 | 36 | Male | 21.98 | nd | 5,39,016 | 4,42,944 | 29792 | 99.63 | GRA | BA.2.10 |
| 75 | EPI_ISL_12155387 | 24 | Female | 16.86 | na | 1,16,956 | 1,15,471 | 29606 | 99.01 | GRA | BA.2.10 |
| 76 | EPI_ISL_12155388 | 19 | Male | 23.44 | na | 2,80,440 | 2,70,735 | 29482 | 98.59 | GRA | BA.2 |
| 77 | EPI_ISL_12155389 | 26 | Male | 22.13 | New Delhi | 2,54,048 | 2,50,870 | 29574 | 98.90 | GRA | BA.2 |
| 78 | EPI_ISL_12155390 | 21 | Male | 19.63 | Lucknow | 2,57,712 | 2,24,796 | 29577 | 98.91 | GRA | BA.2.10 |
| 79 | EPI_ISL_12155391 | 23 | Male | 25.6 | na | 4,77,166 | 4,19,537 | 29788 | 99.62 | GRA | BA.2.10 |
| 80 | EPI_ISL_12155392 | 32 | Male | 20.64 | na | 3,65,918 | 3,52,749 | 29604 | 99.00 | GRA | BA.1.1.7 |
| 81 | EPI_ISL_12155393 | 55 | Male | 21.21 | nd | 5,19,484 | 5,01,441 | 29770 | 99.56 | GRA | BA.2.10 |
| 82 | EPI_ISL_12155394 | 29 | Male | 28.73 | nd | 2,85,936 | 2,80,149 | 29494 | 98.63 | GRA | BA.2.10 |
| 83 | EPI_ISL_12155395 | 46 | Male | 20.5 | nd | 1,66,598 | 1,64,437 | 29577 | 98.91 | GRA | BA.2.10 |
| 84 | EPI_ISL_12155396 | 48 | Male | 22.05 | nd | 6,12,746 | 4,78,153 | 29767 | 99.55 | GRA | BA.2.10 |
| 85 | EPI_ISL_12155397 | 33 | Male | 18.74 | na | 1,67,408 | 1,65,774 | 29738 | 99.45 | GRA | BA.2.10 |
| 86 | EPI_ISL_12155398 | 60 | Male | 21.41 | na | 3,29,882 | 3,22,260 | 29620 | 99.05 | GRA | BA.2 |
| 87 | EPI_ISL_12155399 | 19 | Female | 22.32 | na | 2,37,096 | 1,97,128 | 29406 | 98.34 | GRA | BA.2 |
| 88 | EPI_ISL_12155400 | 25 | Female | 17.1 | nd | 1,66,026 | 1,63,500 | 29701 | 99.32 | GRA | BA.1.1.7 |
| 89 | EPI_ISL_12157232 | 47 | Male | 18.44 | na | 2,95,100 | 2,83,789 | 29607 | 99.01 | GRA | BA.2.10 |
| 90 | EPI_ISL_12155401 | 36 | Female | 17.91 | na | 2,09,476 | 2,06,357 | 29734 | 99.43 | GRA | BA.2 |
| 91 | EPI_ISL_12155402 | 36 | Male | 16.51 | na | 1,76,274 | 1,74,109 | 29726 | 99.41 | GRA | BA.2 |
| 92 | EPI_ISL_12155403 | 44 | Male | 16.8 | na | 1,99,204 | 1,96,473 | 29621 | 99.06 | GRA | BA.2.10 |
| 93 | EPI_ISL_12155404 | 28 | Male | 18.66 | na | 2,04,596 | 2,02,005 | 29608 | 99.01 | GRA | BA.2.10 |
| 94 | EPI_ISL_12155405 | 45 | Male | 18.91 | na | 2,07,210 | 2,04,750 | 29515 | 98.70 | GRA | BA.2.10 |
| 95 | EPI_ISL_12155406 | 50 | Male | 19.61 | na | 3,06,616 | 3,02,593 | 29625 | 99.07 | GRA | BA.2 |
| 96 | EPI_ISL_12155407 | 38 | Male | 15.99 | na | 2,14,902 | 2,10,343 | 29598 | 98.98 | GRA | BA.2.10 |
| 97 | EPI_ISL_12155408 | 5 | Male | 20.49 | na | 2,88,942 | 2,85,401 | 29610 | 99.02 | GRA | BA.2.10 |
| 98 | EPI_ISL_12155409 | 30 | Male | 17.69 | na | 2,05,324 | 2,02,756 | 29580 | 98.92 | GRA | BA.2.10 |
| 99 | EPI_ISL_12155410 | 30 | Female | 18.1 | na | 2,23,208 | 1,58,639 | 29536 | 98.77 | GRA | BA.2.10 |
| 100 | EPI_ISL_12155411 | 22 | Male | 18.05 | Gujrat | 3,22,662 | 3,17,083 | 29596 | 98.97 | GRA | BA.2.10 |
| 101 | EPI_ISL_12155412 | 28 | Female | 17.03 | na | 2,08,544 | 2,03,677 | 29651 | 99.16 | GRA | BA.2.10 |
| 102 | EPI_ISL_12155413 | 31 | Female | 23.15 | Hyderabad | 3,49,288 | 3,39,226 | 29600 | 98.99 | GRA | BA.2 |
| 103 | EPI_ISL_12155414 | 40 | Female | 19.77 | na | 2,51,486 | 2,47,058 | 29602 | 98.99 | GRA | BA.2 |
| 104 | EPI_ISL_12155415 | 15 | Male | 17.54 | na | 2,84,640 | 2,79,073 | 29594 | 98.97 | GRA | BA.2 |
| 105 | EPI_ISL_12157233 | 44 | Female | 29.58 | nd | 4,51,884 | 2,92,625 | 29630 | 99.09 | GRA | BA.2.10 |
| 106 | EPI_ISL_12155416 | 50 | Male | 18.05 | na | 2,80,356 | 2,76,639 | 29611 | 99.02 | GRA | BA.2 |
| 107 | EPI_ISL_12155417 | 44 | Male | 19.98 | na | 2,54,246 | 2,47,116 | 29620 | 99.05 | GRA | BA.2 |
| 108 | EPI_ISL_12155418 | 24 | Male | 21.23 | na | 2,59,236 | 2,41,960 | 29530 | 98.75 | GRA | BA.2.10 |
| 109 | EPI_ISL_12155419 | 30 | Male | 17.6 | na | 1,46,762 | 1,45,423 | 29691 | 99.29 | GRA | BA.2.10 |
| 110 | EPI_ISL_12155420 | 17 | Male | 18.55 | Agra | 1,72,466 | 1,70,324 | 29784 | 99.60 | GRA | BA.2.10 |
| 111 | EPI_ISL_12155421 | 50 | Male | 22.15 | nd | 2,15,546 | 2,10,995 | 29605 | 99.00 | GRA | BA.2 |
| 112 | EPI_ISL_12155422 | 23 | Male | 18.33 | na | 2,69,890 | 2,65,403 | 29580 | 98.92 | GRA | BA.2.10 |
| 113 | EPI_ISL_12155423 | 40 | Male | 32.31 | na | 5,78,060 | 5,24,139 | 29650 | 99.15 | GRA | BA.2 |
| 114 | EPI_ISL_12157234 | 25 | Male | 19.64 | nd | 2,29,688 | 2,24,611 | 29581 | 98.92 | GRA | BA.2.10 |
| 115 | EPI_ISL_12155424 | 28 | Female | 19.45 | na | 2,90,250 | 2,87,146 | 29582 | 98.93 | GRA | BA.2.10 |
| 116 | EPI_ISL_12155425 | 75 | Male | 15.38 | New Delhi | 2,76,762 | 2,72,765 | 29792 | 99.63 | GRA | BA.2.10 |
| 117 | EPI_ISL_12155426 | 6 | Male | 19.96 | na | 2,17,194 | 2,14,416 | 29612 | 99.03 | GRA | BA.2 |
| 118 | EPI_ISL_12155427 | 23 | Female | 19.75 | na | 1,95,268 | 1,90,756 | 29569 | 98.88 | GRA | BA.2 |
| 119 | EPI_ISL_12155428 | 60 | Male | 24.66 | nd | 4,66,996 | 3,31,050 | 29760 | 99.52 | GRA | BA.2.10 |
| 120 | EPI_ISL_12155429 | 18 | Female | 14.72 | na | 1,50,254 | 1,48,047 | 29604 | 99.00 | GRA | BA.2 |
| 121 | EPI_ISL_12155430 | 48 | Male | 26.22 | na | 3,04,314 | 2,99,517 | 29434 | 98.43 | GRA | BA.2 |
| 122 | EPI_ISL_12155431 | 7 | Male | 25.32 | na | 2,37,134 | 2,33,756 | 29577 | 98.91 | GRA | BA.2 |
| 123 | EPI_ISL_12155432 | 35 | Female | 23.11 | nd | 2,26,146 | 2,23,083 | 29567 | 98.88 | GRA | BA.2 |
| 124 | EPI_ISL_12155433 | 40 | Male | 19.08 | nd | 2,41,722 | 2,37,653 | 29612 | 99.03 | GRA | BA.2.10 |
| 125 | EPI_ISL_12155434 | 35 | Female | 15.28 | na | 1,99,224 | 1,92,889 | 29672 | 99.23 | GRA | BA.2 |
| 126 | EPI_ISL_12155435 | 32 | Female | 23.34 | na | 4,96,074 | 4,56,718 | 29794 | 99.64 | GRA | BA.2 |
| 127 | EPI_ISL_12155436 | 28 | Female | 19.85 | na | 3,26,126 | 2,74,442 | 29713 | 99.36 | GRA | BA.2.10 |
| 128 | EPI_ISL_12155437 | 33 | Female | 17.98 | nd | 4,16,938 | 3,66,838 | 29801 | 99.66 | GRA | BA.2.10 |
| 129 | EPI_ISL_12155438 | 24 | Male | 27.74 | na | 2,96,804 | 2,89,159 | 29531 | 98.76 | GRA | BA.2 |
| 130 | EPI_ISL_12155440 | 25 | Male | 20.2 | na | 3,94,566 | 3,33,475 | 29772 | 99.56 | GRA | BA.2.10 |
| 131 | EPI_ISL_12155441 | 25 | Male | 18.27 | na | 5,55,332 | 5,27,144 | 29799 | 99.65 | GRA | BA.2.10 |
| 132 | EPI_ISL_12155442 | 25 | Male | 20.53 | na | 4,29,602 | 3,93,283 | 29802 | 99.66 | GRA | BA.2 |
| 133 | EPI_ISL_12155443 | 26 | Male | 25.4 | na | 3,57,206 | 3,05,320 | 29598 | 98.98 | GRA | BA.2 |
| 134 | EPI_ISL_12155444 | 31 | Male | 20.32 | na | 4,32,116 | 3,92,429 | 29828 | 99.75 | GRA | BA.1.1 |
| 135 | EPI_ISL_12155445 | 23 | Male | 18.21 | nd | 4,43,148 | 3,70,755 | 29792 | 99.63 | GRA | BA.2 |
| 136 | EPI_ISL_12155446 | 32 | Male | 20.08 | na | 4,48,524 | 3,56,487 | 29749 | 99.49 | GRA | BA.2.10 |
| 137 | EPI_ISL_12155447 | 51 | Male | 26.73 | nd | 3,65,596 | 3,54,931 | 29518 | 98.71 | GRA | BA.2.10 |
| 138 | EPI_ISL_12155448 | 28 | Male | 26.93 | na | 3,84,714 | 3,73,623 | 29728 | 99.41 | GRA | BA.2 |
| 139 | EPI_ISL_12155449 | 23 | Female | 15.26 | nd | 3,26,810 | 3,01,815 | 29802 | 99.66 | GRA | BA.2.10 |
| 140 | EPI_ISL_12155450 | 25 | Male | 24.94 | na | 3,20,150 | 2,96,993 | 29361 | 98.19 | GRA | BA.2.10 |
| 141 | EPI_ISL_12155451 | 24 | Male | 24.18 | na | 3,10,216 | 2,92,043 | 29791 | 99.63 | GRA | BA.2.12 |
| 142 | EPI_ISL_12155452 | 24 | Male | 18.5 | na | 4,27,600 | 3,71,880 | 29799 | 99.65 | GRA | BA.2 |
| 143 | EPI_ISL_12155453 | 32 | Male | 21.14 | na | 2,39,948 | 2,14,171 | 29795 | 99.64 | GRA | BA.2 |
| 144 | EPI_ISL_12155454 | 34 | Male | 24.4 | na | 3,07,558 | 2,43,372 | 29763 | 99.53 | GRA | BA.2.10 |
| 145 | EPI_ISL_12155455 | 36 | Female | 23.38 | na | 4,02,434 | 3,36,535 | 29697 | 99.31 | GRA | BA.2.10 |
| 146 | EPI_ISL_12155456 | 30 | Female | 27.44 | Mumbai | 4,29,554 | 2,70,637 | 29278 | 97.91 | GRA | BA.1.18 |
